# Dual isolation of primary neurons and oligodendrocytes from guinea pig frontal cortex

**DOI:** 10.3389/fncel.2023.1298685

**Published:** 2024-01-10

**Authors:** Roisin A. Moloney, Carlton L. Pavy, Richard G. S. Kahl, Hannah K. Palliser, Jon J. Hirst, Julia C. Shaw

**Affiliations:** ^1^School of Biomedical Sciences and Pharmacy, University of Newcastle, Newcastle, NSW, Australia; ^2^Hunter Medical Research Institute, Mothers and Babies Research Centre, Newcastle, NSW, Australia

**Keywords:** neuron, oligodendrocyte, primary cell culture, guinea pig, frontal cortex

## Abstract

Primary cell culture is a technique that is widely used in neuroscience research to investigate mechanisms that underlie pathologies at a cellular level. Typically, mouse or rat tissue is used for this process; however, altricial rodent species have markedly different neurodevelopmental trajectories comparatively to humans. The use of guinea pig brain tissue presents a novel aspect to this routinely used cell culture method whilst also allowing for dual isolation of two major cell types from a physiologically relevant animal model for studying perinatal neurodevelopment. Primary neuronal and oligodendrocyte cell cultures were derived from fetal guinea pig's frontal cortex brain tissue collected at a gestational age of 62 days (GA62), which is a key time in the neuronal and oligodendrocyte development. The major advantage of this protocol is the ability to acquire both neuronal and oligodendrocyte cellular cultures from the frontal cortex of one fetal brain. Briefly, neuronal cells were grown in 12-well plates initially in a 24-h serum-rich medium to enhance neuronal survival before switching to a serum-free media formulation. Oligodendrocytes were first grown in cell culture flasks using a serum-rich medium that enabled the growth of oligodendrocyte progenitor cells (OPCs) on an astrocyte bed. Following confluency, the shake method of differential adhesion and separation was utilized via horizontally shaking the OPCs off the astrocyte bed overnight. Therefore, OPCs were plated in 12-well plates and were initially expanded in media supplemented with growth hormones, before switching to maturation media to progress the lineage to a mature phenotype. Reverse transcription-polymerase chain reaction (RT-PCR) was performed on both cell culture types to analyze key population markers, and the results were further validated using immunocytochemistry. Primary neurons displayed the mRNA expression of multiple neuronal markers, including those specific to GABAergic populations. These cells also positively stained for microtubule-associated protein 2 (MAP2; a dendritic marker specific to neurons) and NeuN (a marker of neuronal cell bodies). Primary oligodendrocytes expressed all investigated markers of the oligodendrocyte lineage, with a majority of the cells displaying an immature oligodendrocyte phenotype. This finding was further confirmed with positive oligodendrocyte transcription factor (OLIG2) staining, which serves as a marker for the overall oligodendrocyte population. This study demonstrates a novel method for isolating both neurons and oligodendrocytes from the guinea pig brain tissue. These isolated cells display key markers and gene expression that will allow for functional experiments to occur and may be particularly useful in studying neurodevelopmental conditions with perinatal origins.

## 1 Introduction

Neurons and oligodendrocytes are two key cell types, which allow for the receipt, production, flow, and processing of information in the brain. Neurons are responsible for key chemical signaling throughout the brain, while oligodendrocytes play a pivotal role in supporting neuronal signaling through myelination (Bradl and Lassmann, 2010). Myelination is critically important for neuronal conductance as myelin sheaths are responsible for insulating axons, thereby increasing the speed and efficiency of neuronal signaling. Oligodendrocytes develop and differentiate through a lineage of maturational stages before finally becoming mature oligodendrocytes that are responsible for the synthesis of myelin in the fetal and neonatal brain (Jakovcevski et al., 2009). Oligodendrocytes begin as mitotically active cells knows as oligodendrocyte progenitor cells (OPCs) before progressing into the immature oligodendrocyte stage (Back et al., 2001, 2002). Following the immature oligodendrocyte stage, oligodendrocytes mature through the late developmental stages of pre-myelinating and mature myelinating oligodendrocytes (Giacci and Fitzgerald, 2018). Oligodendrocytes are particularly susceptible to excitotoxic damage, particularly when exposed to high levels of glutamate and reactive oxygen species (ROS) (Kuhn et al., 2019). Many neurodevelopmental pathologies stemming from birth-related insults involve periods of hypoxia and related increases in excitation, highlighting the importance of understanding the effects on oligodendrocyte maturation and, consequently, myelination. Damage or aberration to neurons or oligodendrocytes is seen in almost all neurological disorders, and therefore, it is important to study the relationship between these cell types in a clinically translational model.

In this article, we will outline the protocol by which primary neurons and oligodendrocytes can be isolated and cultured from the frontal cortex of fetal guinea pigs. Guinea pigs exhibit a pregnancy hormone profile more similar to humans than rats and mice, and of particular importance is the sustained production of progesterone by the placenta until term. Progesterone is known to be a key substrate in the production of many neurosteroids, which play vital roles in neurodevelopment, therefore allowing a more translationally accurate model to study the prenatal and early postnatal brain development (Hirst et al., 2018; Mullins et al., 2020). Guinea pigs, unlike rats and mice, are a highly precocial species, with most of their brain development occurring prior to birth, which is more comparable to humans. In contrast, rats undergo rapid myelination following birth, reaching 50% of myelination at ~4 weeks after birth, and myelination is considered complete at ~8 weeks after birth (Watson et al., 2006; Han et al., 2022). Guinea pigs, being a precocial species, have a higher proportion of axonal myelination and maturation at birth compared to altricial species due to the prenatal gliogenesis and myelination. These differences make this species a more translationally relevant model for preclinical studies of human fetal and neonatal brain development (Kalusa et al., 2021).

This protocol allows for the concurrent isolation of neurons and oligodendrocytes from the same brain across multiple regions, minimizing tissue wastage for each animal and allowing for a direct comparison of different cell types from the same animal. The unique adherent properties and cell sizes of different central nervous system (CNS) cell types are used to isolate neurons and oligodendrocytes. This protocol is a cost-effective method whereby the primary tools used to isolate the neurons and oligodendrocytes are cell strainers and mechanical shakers, both of which are commonplace in many laboratory settings. The reagents utilized to maintain these cultures are standard and widely used in cell culture, making this protocol a highly effective tool to study these key cell types.

The dual isolation of primary neurons and oligodendrocytes from the fetal guinea pig's frontal cortex has not been detailed elsewhere; however, this protocol expands on a previous guinea pig neuronal culture protocol (Cohen et al., 2000). Currently, there are no protocols detailing the isolation of oligodendrocyte cells from guinea pig tissue; therefore, this protocol was constructed based on previous oligodendrocyte protocols derived from rat tissues (McCarthy and de Vellis, 1980). The temporal similarity between guinea pig and human neurodevelopment *in utero* highlights the enhanced translational relevance of this preclinical model, especially for perinatal neurodevelopment in comparison with the more traditionally used altricial rat and mouse animal models.

## 2 Methods

### 2.1 Animals

Before beginning this research, approval for all the animal experiments and procedures carried out throughout this study was obtained from the University of Newcastle Animal Care and Ethics Committee (A-2020-102). In addition, all experiments and procedures were carried out in accordance with the National Health and Medical Research Council Australian Code of Practice for the Care and Use of Animals for Scientific Purposes. Tri-color outbred female guinea pig dams were obtained from the University of Newcastle Animal Services Unit. Dams were time-mated post-partum, housed indoors under a 12-h light/dark cycle, and were supplied with a diet consisting of commercial guinea pig pellets, hay, and fresh vegetables. Dams were euthanized via CO_2_ inhalation at a gestational age of 62 days. For this study, seven dams were used, which birthed eight male pups and seven female pups.

### 2.2 Cell culture protocol

**Figure 1:**
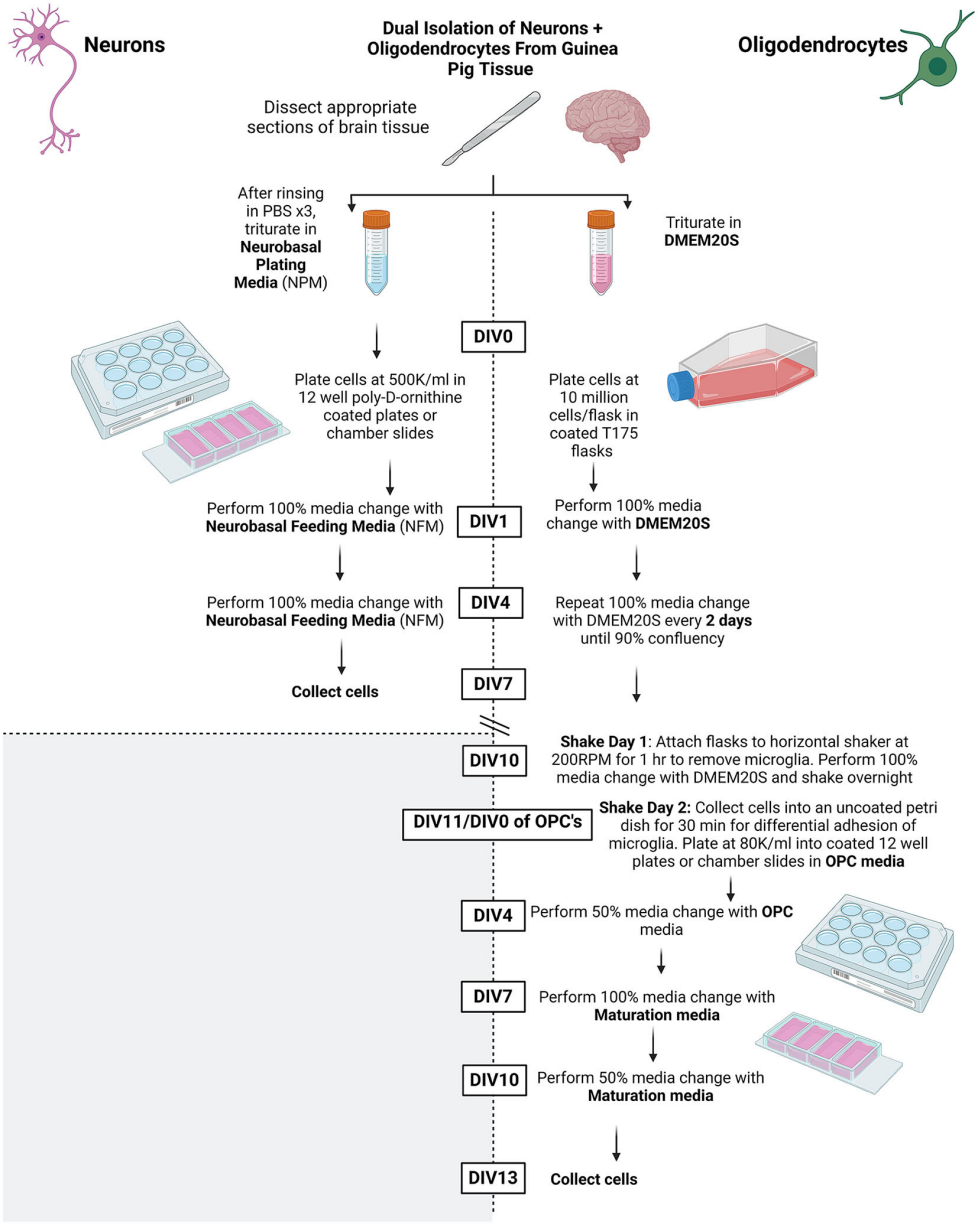
Summary schematic of methods protocol. Created using BioRender.com.

Refer to Figure 1 for overall summary.

#### 2.2.1 Preparation (1 day prior) steps

For the preparation, poly-D-lysine coated flasks or poly-D-ornithine coated plates were used.

1. Dilute the stock of poly-D-lysine or poly-D-ornithine [1 mg/ml in Dulbecco's phosphate-buffered saline (DPBS)] to a working concentration (50μg/ml) with 1 × DPBS and filter sterilize (0.22 μm).
2. Add sufficient quantity of the working concentration solution to cover the surface of flasks, culture plates, or chamber slides and incubate them overnight at 37°C in a cell culture hood.
3. Remove the coating solution, wash with sterile PBS three times, and use immediately or seal with the parafilm and store for up to 1 week.

Media Preparation required for collection: Refer to Tables 1 and **3** for recipes on.

- Neurobasal plating media,
- DMEM20S, and
- Dissecting medium.

**Table 1:** Reagents for cell culture.

	**Name**	**Company and product code**
Media	Dulbecco's modified Eagle's media (DMEM) (high glucose, pyruvate, l-glutamine)	Gibco 11965092
	Dulbecco's modified Eagle's media (DMEM) (no glucose, no pyruvate, l-glutamine)	Gibco 11966025
	Hank's Balanced Salt Solution (HBSS)	Gibco 14175095
	Dulbecco's Phosphate Buffered Solution (DPBS)	Gibco 14040133
	Neurobasal A Media	Gibco 10888022
Growth factors + additives	Papain	Worthington LK003178
	Heat inactivated Horse serum	Merck H1138100ML
	Antibiotic-Antimycotic	Gibco 15240062
	Fetal bovine serum	Bovogen SFBS-F
	HEPES	Gibco 15630080
	Glutamax	Gibco 35050061
	Sodium Pyruvate	Gibco 11360070
	Bovine serum albumin (BSA)	Sigma a79061100G
	DNase	Sigma d5025375ku
	Recombinant human Fibroblast Growth Factor Basic (FGFb)	Gibco 2299059
	Human Apo-transferrin (APO-T)	Merck T1147500MG
	Insulin	Sigma SLCJ8080
	Recombinant Human Platelet-Derived Growth Factor (PDGF)	Australian BioSearch 773704
	Recombinant Human Epidermal Growth Factor (EGF)	Gibco 2209660
	Triiodothyronine (T3)	Merck T2877100MG
Plating reagents	Poly-L-ornithine	Sigma P0421100MG
	Poly-D-lysine	Sigma P08991G

### 2.3 Collection of tissues

#### 2.3.1 Setup

1. Prepare the hood before the collection of tissue with the following supplies (refer to Table 2 for details):
  - Ethanol spray and wipe,
  - Bench pads,
  - Spray tools with 70% ethanol and place open on pads,
  - Waste bucket,
  - Pipette guns,
  - 10 ml serological pipettes,
  - 1,000 μl pipette and tips,
  - Scalpels (2 per pup),
  - Weigh boats, and
  - Paper towel.
2. Prepare papain (refer to Table 3) and activate it in water bath at 37°C. Note: Activate papain at least 15 min before use.
3. Defrost DNase on ice. Note: DNase is sensitive to physical denaturation, so avoid vortexing. Instead, gently invert the tube to mix.
4. Place NPM and DMEM20S into a water bath at 37°C.
5. Place UV hoods.

**Table 2:** Equipment and supplies.

	**Name**	
Tools for dissection	Bee Bee Bone Scissors	
	Surgical Blade	
	Small Blunt Tip Forceps	
	Laboratory Weight Boat	
Tools for isolation	**Name**	**Company and product code**
	CO_2_ Resistant Horizontal Shaker	Life Technologies 88881102
	Cell strainers (40 and 70 μM)	Interpath 542040 and 542070 respectively
	Uncoated petri dish	
	Nunc™ Lab-Tek™ II Chamber Slide™ System (8 well)	Life Technologies 154534

**Table 3:** Media recipes for neuronal and oligodendrocyte isolations.

Dissecting media	500 ml DMEM 10 mM HEPES 1% antibiotic-antimycotic
Neurobasal plating media (NPM)	Neurobasal media 2% B27 supplement—Note: Add B27 as close to the collection as possible. Avoid using medium with B27 after 1 week for enhanced viability. 1% GlutaMax. Note: GlutaMax was superior to standard L-glutamine for preserving cell viability 1% Antibiotic-antimycotic 1 mM HEPES 10% Heat Inactivated Horse Serum
Neurobasal feeding media (NFM)	Neurobasal media 2% B27 supplement—Note: add B27 as close to collection as possible. Do not use medium with B27 after 1 week. 1% GlutaMax. Note: GlutaMax is superior to standard L-glutamine for preserving cell viability 1% Antibiotic antimycotic 1 mM HEPES
DMEM20S	DMEM 20% Fetal bovine serum 1% antibiotic antimycotic
Oligodendrocyte base media	DMEM 0.1% BSA 50 μg/mL Apo-transferrin 5 μg/mL insulin 1% antibiotic antimycotic 30 nM sodium selenite 10 nM D-biotin 10 nM hydrocortisone
OPC media	Oligodendrocyte Base Media 20 ng/mL PDGF-AA 20 ng/mL bFGF 20 ng/ml EGF
Maturation media	Oligodendrocyte Base Media 20 μg/mL triiodothyronine (T3)
Papain preparation	5 ml HBSS 1 vial of papain (the final concentration of 20 U/ml)

#### 2.3.2 Guinea pig pup collection: performed outside of a sterile hood

1. Cull postpartum time-mated dams at approximately GA62 via CO_2_ inhalation, ensuring the absence of reflexes before proceeding.
2. Make an incision down the abdomen and expose the uterus.
3. Using scissors, carefully remove pups from the uterus and then from the amniotic sac, ensuring the absence of reflexes. Determine and record sex of pups.
4. Heavily spray pups with 70% ethanol and decapitate.
5. Place heads in an ice cold PBS for transfer to the sterile hood.

#### 2.3.3 Dissection: performed in a sterile hood

**Figure 2:**
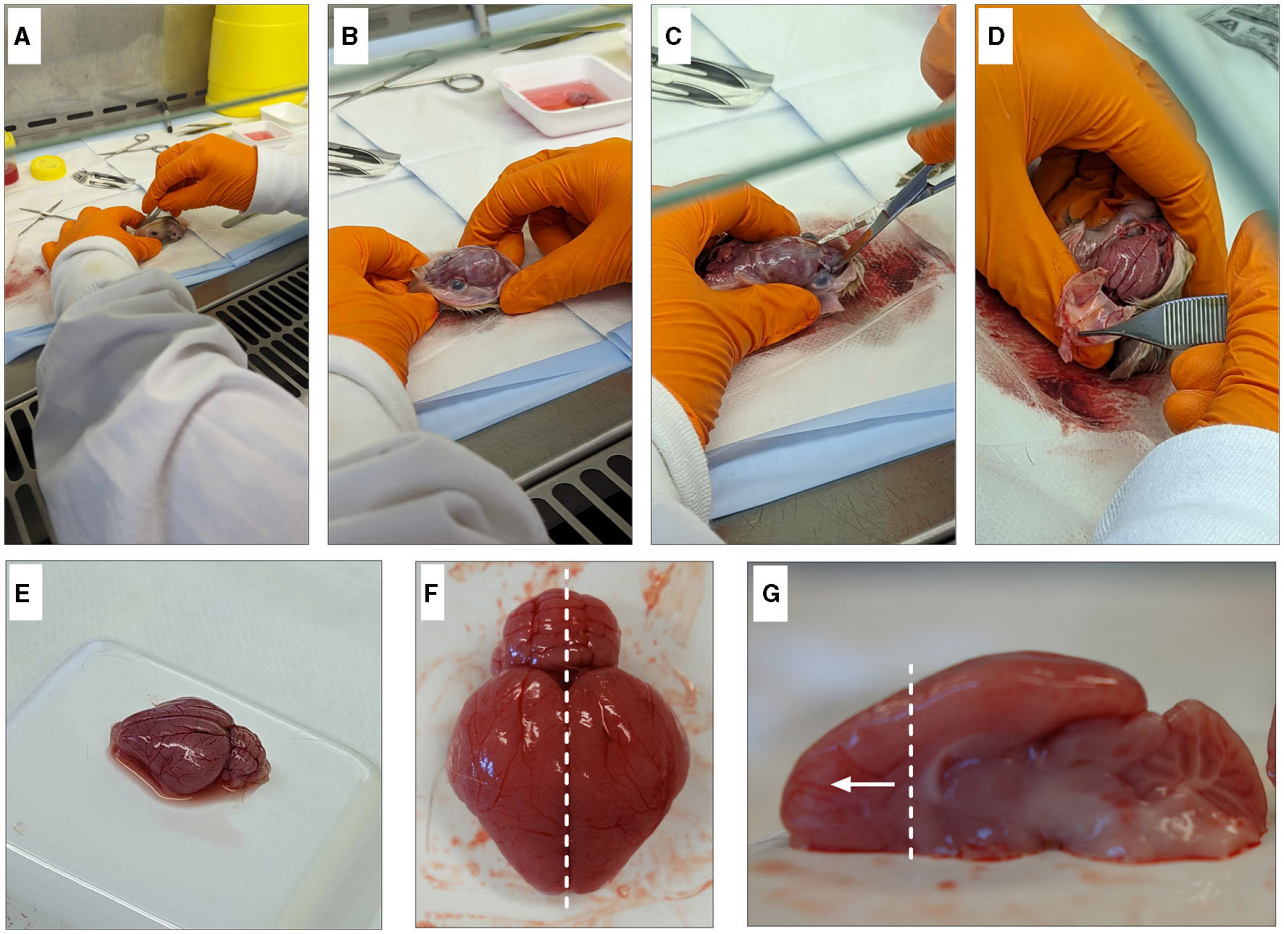
Dissection of guinea pig frontal cortex. Begin by degloving the scalp with a scalpel and exposing the skull **(A, B)**. Cut the bridge of the skull between the eyes **(C)** before cutting up either side to expose the brain **(D)**. Gently remove the brain from the head **(E)** and remove any blood vessels and/or meninges. Dissect down the sagittal plane **(F)** and isolate the frontal cortex **(G)**.

1. Set up weigh boats containing room temperature dissection media. Note: As following the steps require incubation of the tissue in a water bath, avoid placing the tissue directly into the ice cold media to avoid cell death.
2. Remove heads from the ice cold PBS and dry off excess solution; then, spray heavily with 70% ethanol.
3. Make an incision down the midline of the scalp from the nose to the base of the skull and pull back skin to expose the skull (Figures 2A, B).
4. Using scissors, cut the skull along the bridge of the nose. Carefully, cut along each side of the skull, making sure not to damage the brain and peel off the skull (Figures 2C, D).
5. Using tweezers, gently pry the brain out while ensuring that meninges are removed, and the optic nerve is severed to release the brain from the skull without damage. Rinse well to remove any remaining blood.
6. Place each brain immediately into a weigh boat containing dissecting media while collecting the remainder of the brains (refer to Tables 1 and 3 for recipes).
7. To isolate the frontal cortex, begin by separating the hemispheres along the sagittal plane. Using the corpus callosum as a visual guide, isolate the frontal cortex section (Figures 2F, G). Note: One pup's brain is sufficient to produce cultures for four 12-well plates of both neurons and oligodendrocytes.

### 2.4 Neuronal isolation

1. Prepare falcon tubes containing the following per head: 1 ml of a papain mixture (20 U/ml), 1 ml DNase (0.2 mg/ml stock), and 5 ml HBSS.
2. Mince brain sections into 1 mm^3^ chunks and split half of the homogenized mixture into each tube.
3. Invert and ensure that all tissues are covered by a liquid before placing them in a 37°C water bath and gently rocking for 15 min. Invert the tube for every 5 min.
4. To inactivate papain, add 1.5 ml of NPM to the tube.
5. Spin down the tube for 5 min at 270 g and remove the supernatant.
6. Add 5 ml of HBSS to the tube and gently invert the tube before placing it in a rocking water bath to wash the cell pellet. Spin the tube at 270 g and remove the supernatant.
7. Repeat this step a total of three times.
8. Add 10 ml of the warmed NBP media to the cell pellet and use a 10-ml serological pipette to gently pipette up and down ~10 times or until the liquid is homogenous. Avoid excessive trituration or creating bubbles as the cells are very delicate.
9. Let cell suspension settle for 5 min and then pass the supernatant through a 40-μm cell strainer.
10. To the remaining the cell pellets, add another 10 ml of warmed NPM and repeat before passing all remaining liquid through the same strainer as above.
11. Count the cells and plate at 500 K/ml in coated 12-well plates or chamber slides.
12. After 24 h of plating, remove media and gently wash with warmed PBS to remove debris. Replace with NBF. Note: While neurons require initial serum supplementation, a serum-rich medium also promotes astrocytic growth. Therefore, the remainder of the protocol will utilize feeding media, which does not contain horse serum.
13. On DIV4, rinse cells again with warmed PBS to remove debris and replace with NBF. Note: If cultures still contain a high quantity of cell debris, a subsequent rinse with warmed NBF is sufficient to remove the remaining debris without harming cells.
14. The expected yield for this protocol is four 12-well plates of neurons seeded at 500 K/ml or 24 million cells total.
15. On DIV7, collect cells.

### 2.5 Oligodendrocyte isolation

This method can be broken into three key stages: (1) OPC development, (2) OPC expansion, and (3) OPC maturation.

#### 2.5.1 OPC development

1. Prepare falcon tubes containing the following per brain: 1 ml of the papain mixture (20 U/ml), 1 ml of DNase (0.2 mg/ml stock), and 5 ml of HBSS.
2. Mince brain sections into 1 mm^3^ chunks and split half of the homogenized mixture into each tube.
3. Invert and ensure that tissues are covered by the liquid before placing them in a 37°C water bath and gently rocking them for 15 min. Invert the tube for every 5 min.
4. To inactivate papain, add 5 ml of DMEM20S to the tube.
5. Spin down the tube for 5 min at 270 g and remove the supernatant.
6. Add 10 ml of warmed DMEM20S to the cell pellet.
7. Use a 10-ml serological pipette to slowly pipette up and down 10 times or until the liquid is almost homogeneous.
8. Let the liquid settle for a couple of minutes and then pass the supernatant through a 70-μm cell strainer. Note: The use of a larger strainer allows for the inclusion of astrocytes into the cell pool, which will form the supportive bed that the OPCs will grow on. DMEM20S contains fetal bovine serum that facilitates the growth of astrocytes and allows OPCs to develop.
9. Add another 10 ml of warmed DMEM20S to the cell pellet and repeat the process until there is a combined 20 ml of cell suspension.
10. Count cells and plate them at 500 K/ml in 20 ml in a coated T175 flask. Note: Two flasks from each animal will generate enough OPCs for four 12-well plates.
11. After 24 h of plating, remove media and gently wash with warmed PBS. Replace with 20 ml of DMEM20S.
12. Every 2–3 days, perform 100% media change with fresh DMEM20S.
13. At approximately DIV10, flasks should be ~80%−90% confluent. Note: OPCs will be bright round circles that place it on top of a bed of astrocytes when viewed on a light microscope.
14. Attach flasks to a horizontal shaker situated in a 37°C standard cell culture incubator at 200 rpm for 1 h to remove microglia and residual astrocytes.
15. Remove flasks and perform 100% media change with fresh DMEM20S.
16. Seal flask caps with parafilm. Note: A low oxygen environment helps to displace OPCs.
17. Return flasks to a horizontal rocker and leave on 200 rpm for 18 h. Note: Avoidance of leaving for longer as excessive shaking can cause cell death.

#### 2.5.2 OPC expansion

1. Following 18-h shake, remove flasks from the shaker, and in a sterile hood, gently tap sides a couple of times to loosen any remaining OPCs.
2. Collect cell suspension and place it onto a large uncoated petri dish and place it in a standard cell culture incubator for 30 min to allow for the remaining microglia to adhere.
3. Gently swirl the dish and collect it through a 40-μM strainer into a falcon tube.
4. Centrifuge the falcon tube for 10 min at 300 g.
5. Remove the supernatant with a serological pipette. Note: Excessive caution is required not to disturb cell pellet as it is in a loose condition.
6. Replace cell suspension with 10 ml of OPC media and very gently pipette up and down to dispense the cell pellet into the solution. Note: OPC media contains the growth factors PDGF, FGF, and EGF, which are critical for the expansion of OPCs. Additionally, FBS has been removed from the remainder of the experiment as it encourages astrocytic growth.
7. Count cells and plates at 80 K/ml in coated 12-well plates or chamber slides.
8. Change 50% of the media on DIV3.

#### 2.5.3 OPC maturation

1. On DIV7, perform a 100% media change and replace it with maturation media. Note: Growth factors in OPC media inhibit maturation, whereas supplementation with T3 is beneficial in differentiating cells into a more mature form.
2. On DIV10, perform a 50% media change.
3. Collect cells at DIV13.
4. The expected yield for this protocol is four 12-well plates of oligodendrocytes seeded at 80 K/ml, or 4 million cells in total.

### 2.6 Collection of neurons and oligodendrocytes

#### 2.6.1 Collection of cell lysate

1. For this study, plate both neuronal and oligodendrocyte cultures in triplicate wells before pooling for collection.
2. Prepare ice cold PBS and the lysis buffer according to the manufacturer's instructions (see below).
3. Rinse plates with ice-cold PBS before adding the lysis buffer into wells.
4. Scrape cells off with a P1000 tip and collect them into Eppendorf tubes.
5. Snap freeze in liquid nitrogen to perform RNA extraction immediately or store at −80°C.

#### 2.6.2 Fixing of chamber slides

1. Aspirate media off chamber slides and rinse with ice-cold PBS.
2. Aspirate PBS and fix cells in 4% paraformaldehyde in PBS for 10 min at room temperature.
3. Replace with PBS/sodium azide (0.05%) for a long-term storage.

### 2.7 Characterization of cell populations

#### 2.7.1 Real-time PCR

RNA was extracted using the Bioline Isolate II Micro RNA kit (Meridian Bioscience, Ohio, USA), as per manufacturer's instructions. Briefly, cells were collected into lysis buffer supplied with reducing agent tris (2-carboxyethyl) phosphine (TCEP). Carrier RNA (poly-A RNA: poly(A) potassium salt) was added to assist with RNA recovery at a final concentration of 20 ng/sample. Lysate was filtered to remove debris with DNase digestion performed on column. Total RNA was quantified with the Nanodrop™ One Spectrophotometer, where the quality and purity of RNA were determined using A260/A280 and A260/A230 ratios. Further quality assessment was verified by agarose gel electrophoresis. Synthesis of cDNA was performed using the Superscript IV Reverse Transcription kit (Invitrogen, Carlsbad, California) with random hexamers using a GeneAmp 9700 PCR Machine.

Primer sequences were designed and optimized for guinea pigs in the study laboratory and are detailed in Table 4. Gene data expression was obtained using the Fluidigm Juno and Biomark systems, as previously described (Crombie et al., 2021, 2023). First, samples were preamplified using the PreAmp Master Mix according to manufacturer's instructions. Primers were prepared (0.5 pmol/μl) in EVAGreen, and RT-PCR was performed using the Biomark HD system. A calibrator of pooled brain tissue samples was used to ensure consistency between plates. The results were analyzed using the comparative CT method of analysis normalized to the housekeeping genes ACTB, TBP, YWHAZ, and UBE2D2. One sample of frontal cortex brain tissue was used as a positive control, and one sample of placental tissue was used as a negative control.

**Table 4:** Guinea pig-specific primers for real-time PCR.

**Gene ID**	**Protein**	**Forward primer**	**Reverse primer**	**Amplicon size (bp)**
*DRD1*	Dopamine receptor D1	ACCTCCAGCATGGATGAGAC	TGACAGGAAACAGGCTGTCA	78
*DRD2*	Dopamine receptor D2	CCTGCCAAGCCAGAGAAGAA	GGGCATGGACTGGATCTCAAA	78
*GABRA1*	GABAA α1 receptor	CTCAAGCCCGCAATGAAGAAA	TCCAGTCAACGTGCTCAGAA	81
*GABRA2*	GABAA α2 receptor	ACTAGGCCAATCAATTGGGAA	TCAAGTGGAAATGAGCTGTCA	80
*GABRA3*	GABAA α3 receptor	TTGGCAGCTATGCCTACACA	ACCTCCACAGACTTGTTCTTCC	73
*GABRA5*	GABAA α5 receptor	TGGTTCATCGCTGTGTGCTA	CCCAGCCTCTCTTCGTGAAATA	85
*RBFOX3*	RNA binding fox-1 homolog 3 (NeuN)	CACAGACAGACAGCCAACCA	CGGAAGGGGATGTTGGAGAC	88
*SYP*	Synaptophysin	TTCAGGCTGCACCAAGTGTA	GTAGTCCCCAACGAGGAAGAC	78
*INA*	Internexin neuronal intermediate filament protein alpha	ACAAGATCATCCGCACCAAC	GTGCACCTTTTCGATGAACAC	80
*DLG4*	Postsynaptic density protein 95 (PSD-95)	TATTCCCAGCACCTGGACAA	TCATGGCTGTGGGGTAATCA	70
*PVALB*	Parvalbumin	AAGGATGGGGACGGCAAA	GGGTCCATCAGCTCTGCTTA	77
*CALB1*	Calbindin	CTGACTGAGATGGCCAGGTTA	CCCACACATTTTAACTCCCTGAAA	75
*SST*	Somatostatin	AAGCAGGAACTGGCCAAGTA	TGGGACAAATCTTCAGGTTCCA	92
*GRIA1*	Glutamate ionotropic receptor AMPA type subunit 1	TGAACGCAGGACTGTCAACA	AAGCTCGGTGTGATGAAGCA	72
*GRIA2*	Glutamate ionotropic receptor AMPA type subunit 2	GACACCTCACATCGACAACC	CGCCTCTTGAAAACTGGGAA	80
*GRIN1*	Glutamate ionotropic receptor NMDA type subunit 1	AGAGCATCCACTTGAGCTTCC	TACACGCGCATCATCTCGAA	82
*GFAP*	Glial fibrillary acidic protein	AAGAGGCATCCAGCTACCAG	GGTAGGTGGCAATCTCGATGT	81
*OLIG2*	Oligodendrocyte transcription factor	GCACTCATCCTGGGGACAA	CCGACGACGTGGATGATGAA	78
*NCAM1*	Neural cell adhesion molecule 1	TTGTTCCCAGCCAAGGAGAA	TGTCTTTGGCATCTCCTGCTA	78
*CSPG4*	Chondroitin sulfate proteoglycan 4	CTCCTCACCACCACCCTCAA	ACTCTTCAGCACAGCCCTCA	79
*GALC*	Galactosylceramidase	ACTTCCCGCCTTCTGGTAAA	AGGTTCAGTGCCATCTGTTGT	144
*MBP*	Myelin basic protein	ACCTCCTCCGTCTCAAGGAAA	GCTCTGCCTCCATAGCCAAA	66
*ACTB*	Housekeeper	TGCGTTACACCCTTTCTTGAC A	ACAAAGCCATGCCAATCTCAT	72
*YHWHAZ*	Housekeeper	GCTTCACAAGCAGAGAGCAA	CAGCAACTTCGGCCAAGTAA	76
*TBP*	Housekeeper	CAAGCGGTTTGCTGCTGTAA	CACCATCTTCCCGGAACTGAA	79
*UBE2D2*	Housekeeper	CAGTGCTGCGTGTTGTACATA	TGCTAGGAGGCAATGTTGGTA	77

Primer sequences for detection of genes of interest in the guinea pig frontal cortex neuronal and oligodendrocyte cells. Primer sequences are displayed from 5′ to 3′ for forward and reverse primers.

The GFAP primer was unable to be optimized on Fluidigm Delta Gene Assay; therefore, it was performed using standard reverse-transcription PCR, as previously described (Shaw et al., 2015). Briefly, RT-PCR was performed using the QuantStudio 6 Flex RT-PCR system (Applied Biosystems; Life Technologies Pty Ltd, Mulgrave, Australia) for GFAP, with Beta Actin (ACTB) used as a housekeeper. Samples were run in duplicate. PCR products were detected with SYBR Green (Applied Biosystems) and analyzed using the Sequence Detection Software v2.01 (Applied Biosystems). Relative fold change was calculated using the comparative Ct Method (2^−Δ*ΔCt*^). The calibrator as described previously was used to minimize variability.

#### 2.7.2 Immunocytochemistry

Following fixation, formaldehyde was carefully aspirated off, and chamber slides were rinsed with PBS three times for 5 min on a rocker. Slides were then incubated for 10 min in PBS-TritonX (0.1%) before rinsing again with PBS three times for 5 min. Slides were then incubated in blocking serum (10% goat serum, 1% BSA, 0.1% Triton-X in PBS) for 30 min at room temperature. Slides were incubated in primary antibodies (mouse anti-NeuN; 1:1,000; MAB377; Sigma, mouse anti-MAP2; 1:1,000; M9942; Sigma, rabbit anti-Olig2; 1:1,000; AB9610; Sigma; both in 1% BSA/0.1% Triton-X in PBS) overnight at 4°C. The following day slides were washed three times for 5 min with PBS before incubation with secondary antibodies for 1 h at room temperature in the dark (Goat anti-mouse AlexaFluor594; ab1105; Life Technologies, Goat anti-rabbit AlexaFluor488; A1108; Life Technologies, both in 1% BSA/0.1% Triton-X in PBS). The media chamber was removed from the slide as per manufacturer's instructions before mounting the cells with glycerol mounting medium with DAPI (Abcam; ab188804) and carefully placing a cover slip over the top. Nail polish was used to set cover slip onto slide. Images were captured using the Nikon Eclipse Ni fluorescent microscope (Nikon) using 20 × magnification and prepared using ImageJ version 1.47 software (Fuji).

#### 2.7.3 Cytotoxicity assay

Cellular cytotoxicity was analyzed using the CyQUANT™ LDH Cytotoxicity Assay Kit test (Invitrogen, Massachusetts, USA), following manufacturer's instructions. Briefly, supernatant was collected from the neuronal cells at DIV3 and DIV7 and from the oligodendrocyte cells at DIV7 and DIV13. Using the Spectrostar Nano (BMG Labtech, Ortenberg, Germany), 50 μl of the supernatant was transferred to a 96-well plate in triplicate and absorbance was measured at 490 and 680 nm. The maximum LDH released from each cell type was determined per replicate by incubating a subset of cells in lysis buffer for 45 min prior to reading. Cytotoxicity values were determined by subtracting the background signal value (680 nm) from the true LDH value (490 nm) and normalizing to the maximum LDH value, with background subtracted.

### 2.8 Statistical analysis

**Figure 3:**
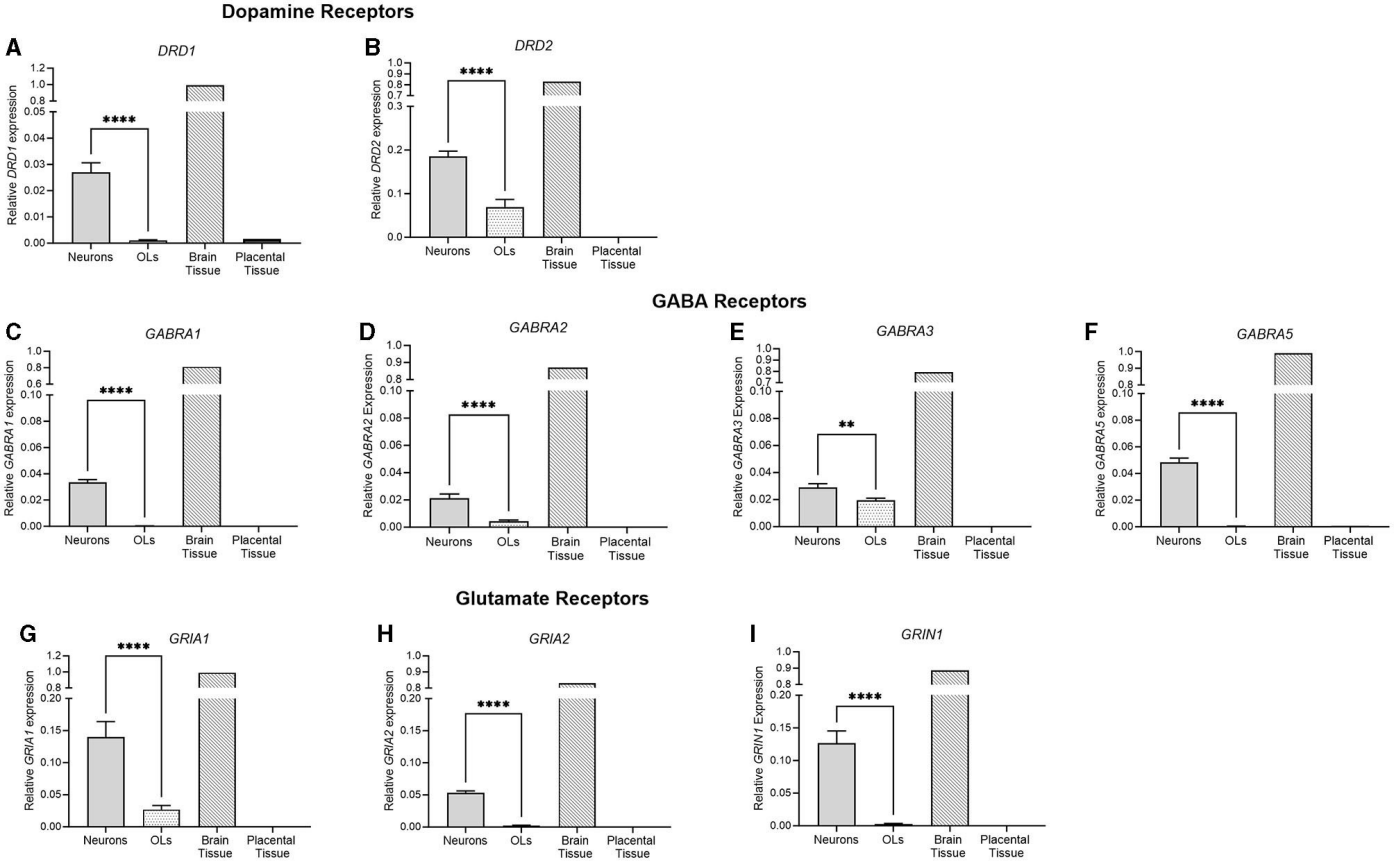
mRNA expression of key dopamine, GABA, and glutamate receptors in primary neurons (light gray bar; *n* = 15), oligodendrocytes (spotted bar, *n* = 15), brain tissue sample (hashed bar; positive control), and placental tissue sample (black bar; negative control). **(A)** *DRD1*, **(B)** *DRD2*, **(C)** *GABRA1*, **(D)** *GABRA2*, **(E)** *GABRA3*, **(F)** *GABRA5*, **(G)** *GRIA1*, **(H)** *GRIA2*, and **(I)** *GRIN1*. Data is presented as mean ± SEM with ***p < 0.001 and ****p < 0.0001.

Data were analyzed using Prism v9.0 (Graphpad Software Inc., La Jolla, California) and presented as mean ± SEM for each group with significance considered at a *p*-value of < 0.05. When neuronal and oligodendrocyte cultures were statistically compared (Figures 3, 8A–G, 9A–F), *t*-tests were performed, with one brain tissue sample acting as a positive control and one placental tissue as a negative control. For groups of three or more (Figures 8H, 9G, H), one-way ANOVAs were performed. For this study, all samples represent an individual biological replicate obtained from one animal. Cells were plated in triplicate per animal and pooled for collection.

## 3 Results

### 3.1 Representative images

**Figure 4:**
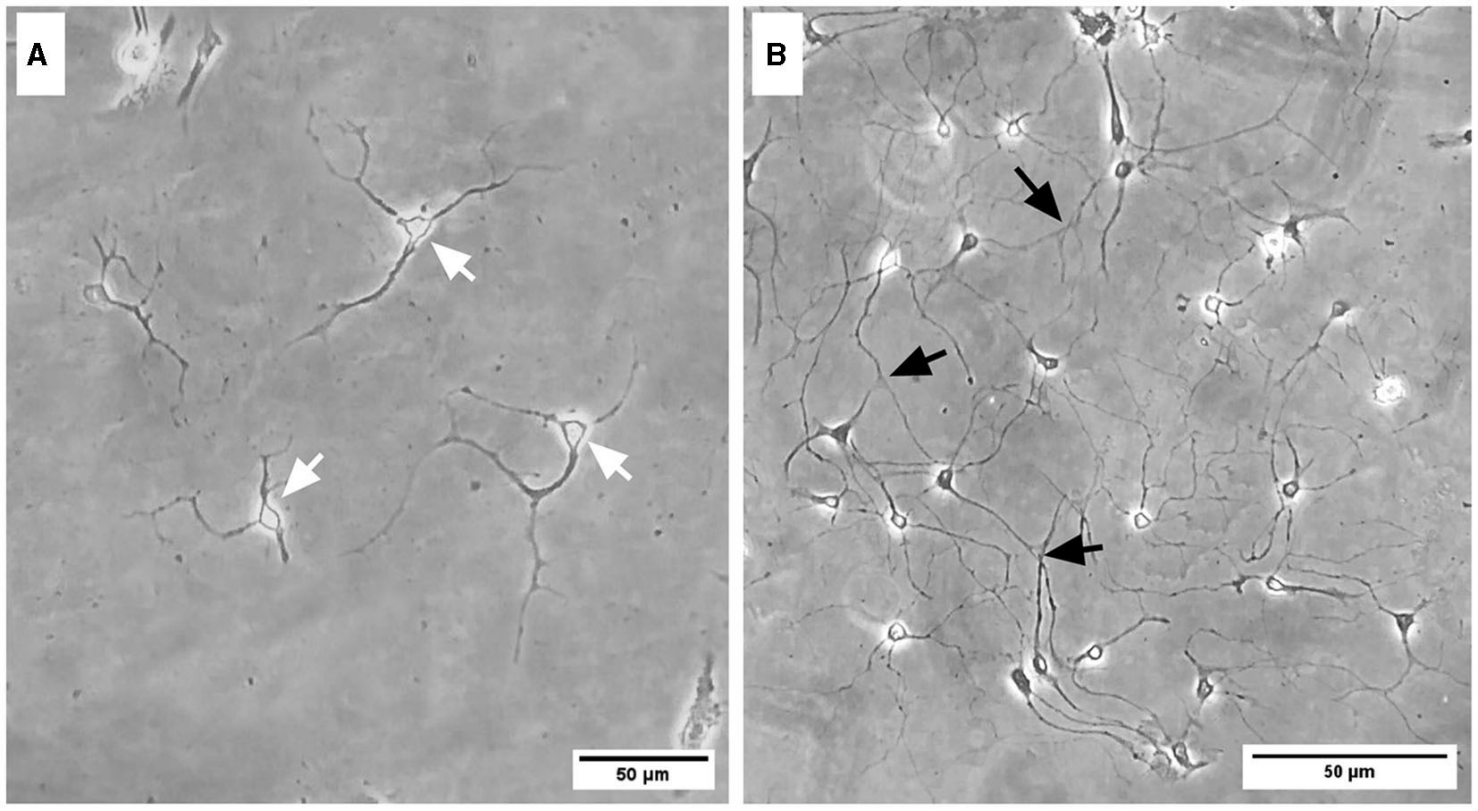
Brightfield microscope images of primary frontal cortex neurons at DIV4 **(A)** and DIV7 **(B)**. White arrows depict immature axonal development, and black arrows indicate synapsing mature neurons. Scale bar = 50 μm.

**Figure 5:**
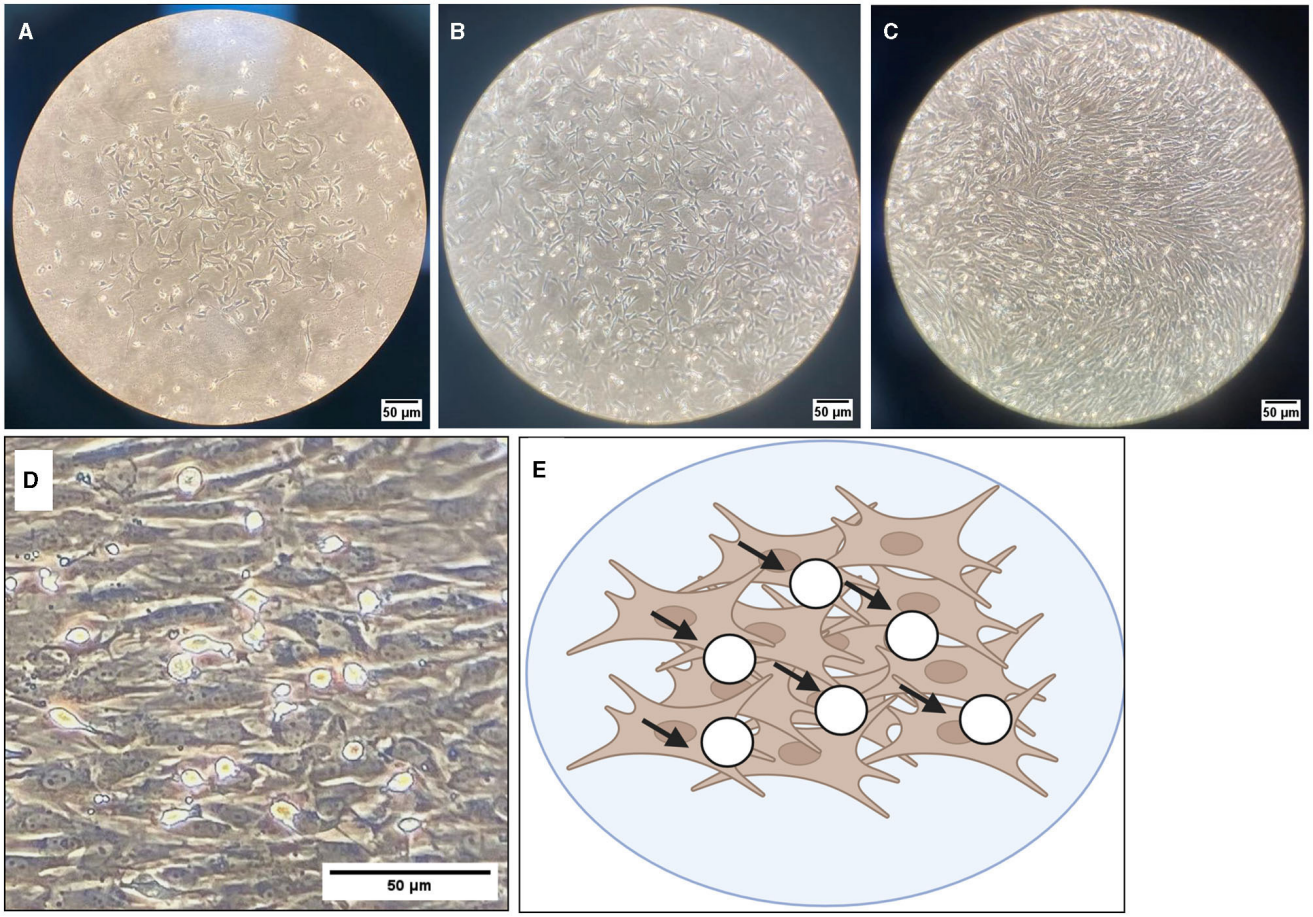
Brightfield microscope images of oligodendrocyte progenitor cells (OPCs; white round circles; black arrows) growing on top of an astrocyte bed (gray cells). Cells are grown in T175 flasks and are shown at 10 × magnification at DIV4 **(A)**, DIV7 **(B)**, and DIV10 **(C)**. **(D)** DIV10 cells at 40 × magnification, with **(E)** showing a graphical representation of OPCs (white circles) on top of the astrocyte bed (brown cells). Scale bar = 50 μm.

**Figure 6:**
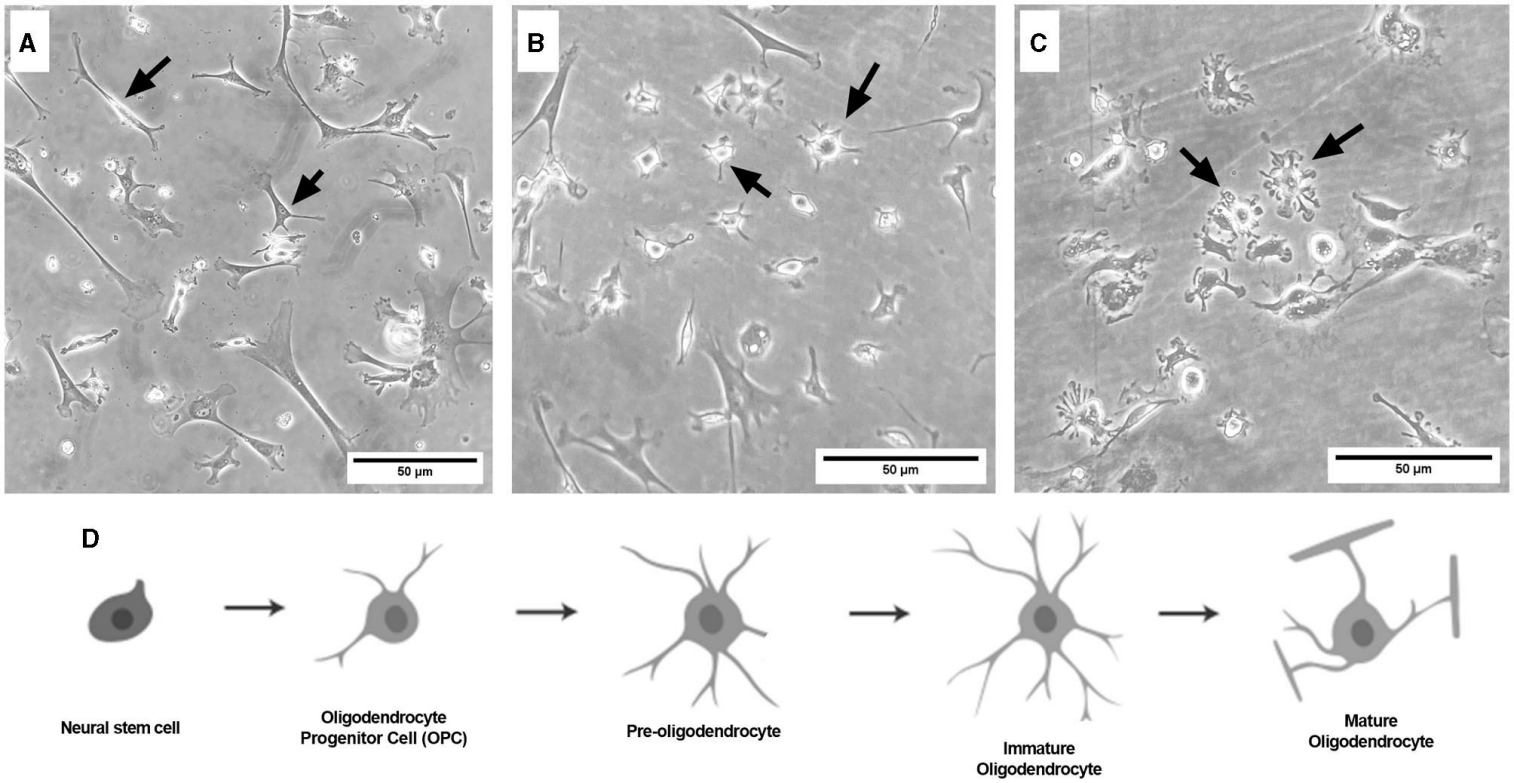
Brightfield microscope images of primary frontal cortex oligodendrocytes at DIV3 [**(A)** OPC stage], DIV7 [**(B)** pre-oligodendrocyte stage], and DIV13 [**(C)** immature oligodendrocyte stage]. Schematic of the oligodendrocyte lineage pathway **(D)**, adapted from Long et al. (2021). Black arrows point to oligodendrocytes at their respective lineage stage. Cells are grown in 12-well plates following the flask stage. Scale bar = 50 μm.

Representative images were taken of primary frontal cortex neurons and oligodendrocytes to show their morphology at different times throughout development. By DIV4, neurons began to develop immature axons (Figure 4A), and by DIV7, synapses and connections formed (Figure 4B). Oligodendrocyte development began in flasks as oligodendrocyte progenitor cells (OPCs; bright white circles) placed on top of a bed of astrocytes (gray flat cells; Figure 5). Initially, the astrocytes were separated with minimal OPC development (Figure 5A), but they formed a solid bed by DIV14, which pushes OPCs to the surface (Figure 5C). Following the overnight shake and plating into 12 well dishes, OPCs began to morphologically change and develop projections (Figure 6).

### 3.2 Immunocytochemistry

**Figure 7:**
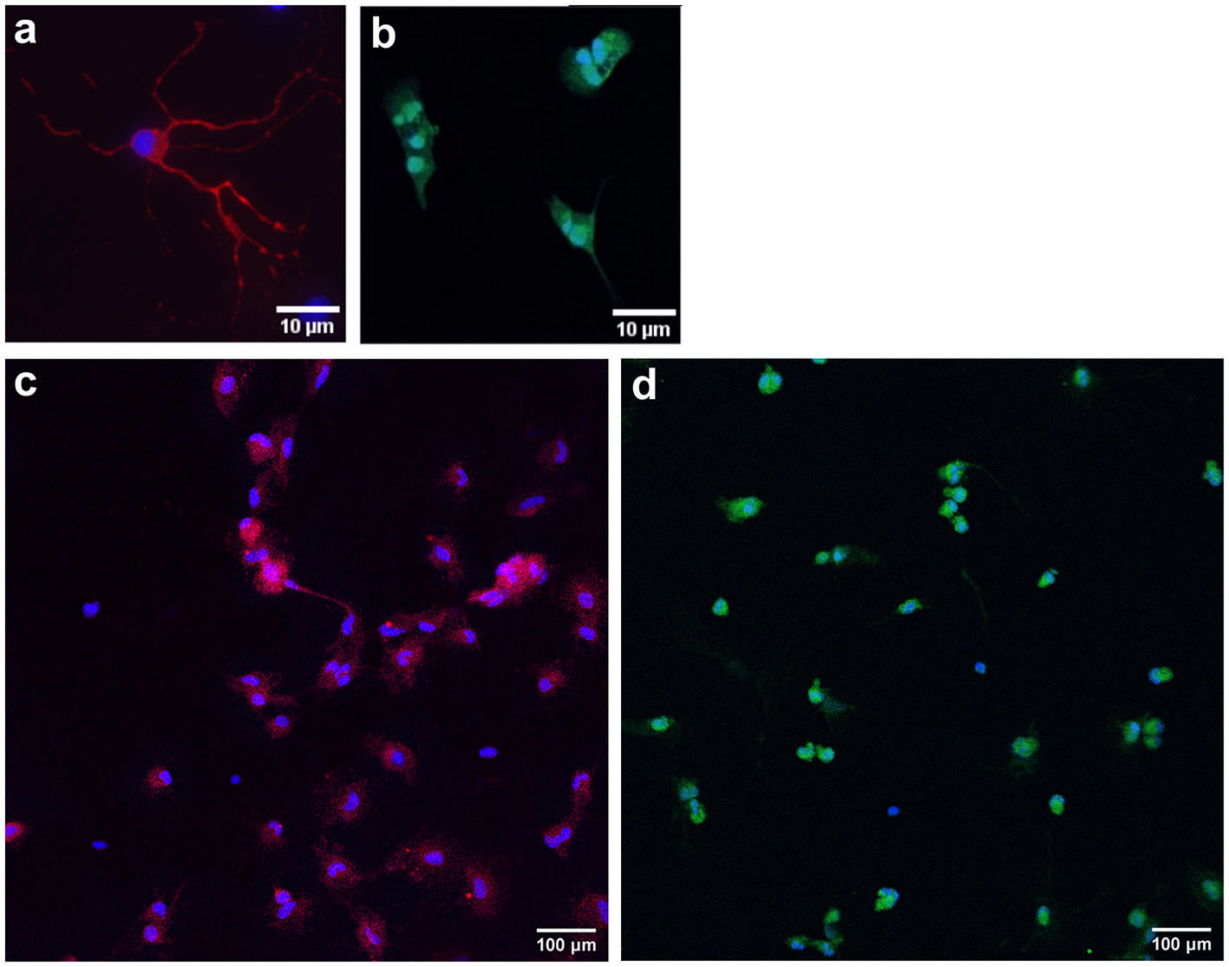
Representative images of immunocytochemistry-stained primary neurons and oligodendrocytes. Primary frontal cortex neurons are stained with MAP2 [red; **(a)**; somatic-dendritic marker] and NeuN [red; **(c)**; cell body marker]. Primary frontal cortex oligodendrocytes are stained with OLIG2 [green; **(b, d)**]. DAPI was used as a nuclear stain (blue). Scale bar = 10 and 100 μm.

To characterize cell populations, immunocytochemistry was performed, and key cell markers were chosen (Figure 7). Neuronal cells were stained with MAP2 (a marker for neuronal somatodendritic compartments of neurons) and NeuN (a marker of neuronal cell bodies). Oligodendrocytes were stained with OLIG2, a marker for overall oligodendrocytes, which stains cell bodies. DAPI was used as a nuclear stain (blue). Neuronal cell cultures were stained with OLIG2, and oligodendrocyte cultures were stained with NeuN to confirm the purity of the populations.

### 3.3 Relative mRNA expression of key population genes

#### 3.3.1 Neuronal markers

**Figure 8:**
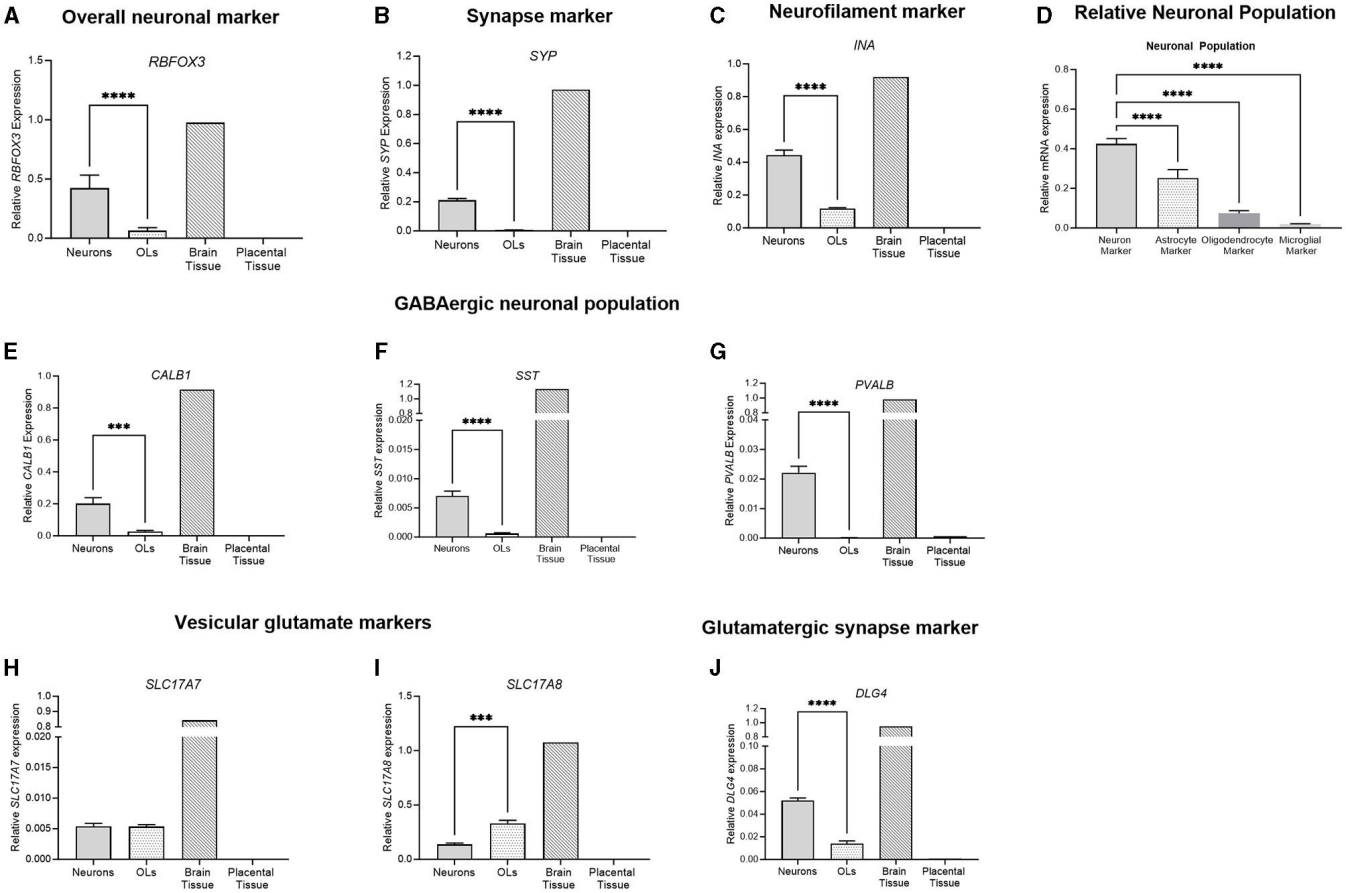
mRNA expression of neuronal markers in primary frontal cortex neurons (light gray bar; *n* = 15), frontal cortex oligodendrocytes (light gray spotted bar, *n* = 15), the brain tissue sample (hashed bar; positive control), and the placental tissue sample (black bar; negative control). Neuronal markers *RBFOX3, SYP* and *INA* were examined **(A–C)**. A comparison was performed between the neuronal marker (*RBFOX3*; light gray column), the astrocyte marker (*GFAP*; spotted column), the oligodendrocyte marker (*OLIG2*; dark gray column), and the microglial marker (*AIF1*; light gray column) to determine the relative population of the cells **(D)**. GABAergic interneurons *CALB1, SST*, and *PVALB* **(E–G)** were quantified, and glutamatergic vesicular markers **(H, I)** and synapse marker *DLG4* **(J)** were also quantified. Data is presented as mean ± SEM with ***p < 0.001 and ****p < 0.0001.

The gene expression of key neuronal genes was examined in both the frontal cortex neuron cell and the oligodendrocyte culture. The brain tissue sample displayed the expression of all genes, while the placental sample displayed no expression (Figure 8). RBFOX3, a marker of overall neuronal expression, had a significantly higher expression in the neuronal samples compared to oligodendrocyte samples (*p* < 0.0001; Figure 8A). SYP is a marker of synaptic terminals, and again, the neuronal culture displays a significantly higher expression compared to oligodendrocyte samples, which had minimal expression (*p* < 0.0001; Figure 8B). α-internexin (INA) is a key neurofilament found in the CNS, and neuronal cultures display a significantly increased expression compared to oligodendrocytes (*p* < 0.0001; Figure 8C). The population of the neuronal cells was determined by comparing the expression of the neuronal marker (RBFOX3), the oligodendrocyte marker (OLIG2), the astrocyte marker (GFAP), and the microglial marker (AIF1). Neuronal cells had a significantly higher expression of RBFOX3 compared to other oligodendrocyte, astrocyte, and microglial markers (*p* < 0.0001 for all), indicating that the population is mostly neuron based (Figure 8D). Components of the GABAergic interneuron population were also examined. The gene expression of three key interneuron genes (*CALB1, SST*, and *PVALB*) was found to be significantly increased in neuronal cell populations compared to oligodendrocytes (*p* < 0.0001 for all; Figures 8E–G). Vesicular glutamate markers VGLUT2 and VGLUT3 (*SLC17A7* and *SLC17A8*) were found to be expressed in both the neuronal and oligodendrocyte populations (Figures 8H, I), where *SLC17A8* was significantly higher in the oligodendrocyte population than in the neuron population (*p* = 0.001). DLG4, a synaptic marker expressed in excitatory neurons, was similarly found to be significantly increased in neuronal cultures compared to oligodendrocytes (*p* < 0.0001).

#### 3.3.2 Oligodendrocyte markers

**Figure 9:**
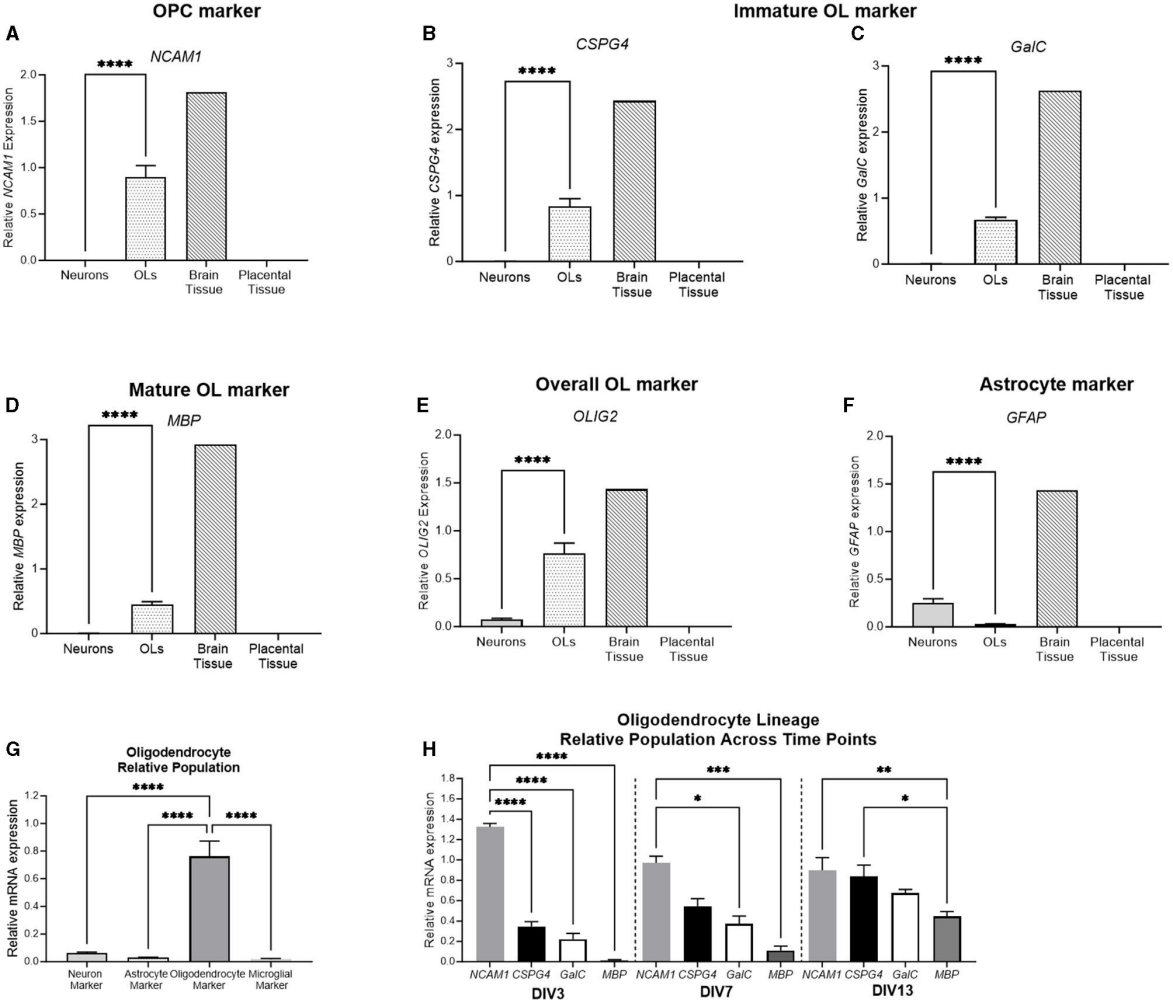
mRNA expression of oligodendrocyte and astrocyte markers in primary neurons (light gray bar; *n* = 15), oligodendrocytes (spotted bar; *n* = 15), brain tissue sample (hashed bar; positive control), and placental tissue sample (black bar; negative control). Oligodendrocyte markers *NCAM1, CSPG4, GalC, MBP*, and *Olig2* **(A–E)**, as well as and astrocyte marker *GFAP* **(F)** were quantified. A comparison was performed between the neuronal (*RBFOX3*; light gray bar), astrocyte (*GFAP*; white bar), oligodendrocyte (*OLIG2*; dark gray bar), and microglial (*AIF1*; lightest gray bar) markers to determine the relative population of the cells **(G)**. **(H)** Delineation of the relative population of oligodendrocyte cells at DIV3, DIV7, and DIV13, with *NCAM1* (striped bar), *CSPG4* (spotted bar), *GalC* (white bar), and *MBP* (dark gray bar). Data is presented as mean ± SEM with *p < 0.05, **p < 0.01, ***p < 0.001, and ****p < 0.0001.

To further delineate the stage of primary oligodendrocyte cells, a range of stage-specific lineage markers were examined (Figure 9). NCAM1 was expressed in early oligodendrocyte precursor cells (OPCs), whereas CSPG4 and GalC were expressed by immature oligodendrocytes (OLs). MBP was expressed by mature OLs, and OLIG2 was expressed by the overall oligodendrocyte population. The relative expression of all oligodendrocyte lineage markers was significantly higher in the oligodendrocyte cell culture than in the neuronal culture (*p* < 0.0001 for all). Additionally, the expression of the astrocyte marker, GFAP, was quantified. GFAP was significantly higher in the neuronal culture than in the oligodendrocyte cell culture (*p* = 0.001), showing that the population primarily involved cells of the oligodendrocyte lineage. The relative population was again delineated by comparing the expression of the four key markers: RBFOX3 (a neuronal marker), OLIG2 (an oligodendrocyte marker), GFAP (an astrocyte marker), and AIF1 (a microglial marker) with the oligodendrocyte culture displaying a significantly higher expression of OLIG2 compared to all other markers (*p* < 0.0001 for all). Based on this finding, the stage-specific lineage markers were also compared at different developmental timepoints. At DIV3, the expression of the early OPC marker NCAM1 was significantly higher than all other oligodendrocyte markers, indicating that the population is undifferentiated (*p* < 0.0001 for all). At DIV7, the expression of NCAM1 was still significantly higher than that of GalC and MBP (*p* = 0.01, *p* = 0.002, respectively). At DIV13, the expression of the early OPC marker NCAM1 (*p* = 0.006) and the immature OL marker CSPG4 (0.02) was significantly higher than that of the mature marker MBP, indicating that the cell population has begun to differentiate into a more mature phenotype.

#### 3.3.3 Receptor expression

The receptor expression of key neurotransmitter systems was also examined. *DRD1* and *DRD2*, the key dopamine receptor subtypes, were both significantly higher in neuronal cells (*p* < 0.0001, *p* < 0.0001), where no DRD1 expression was detected in the oligodendrocyte population. Four of the GABA_A_ subtypes were also examined, and similarly, all four were significantly higher in neuronal cells than oligodendrocytes (*GABRA1*; *p* < 0.0001, *GABRA2*; *p* < 0.0001, *GABRA3*; *p* = 0.0038, *GABRA5*; *p* < 0.0001). No detection was observed in oligodendrocyte cells for *GABRA1* or *GABRA5*. *GRIA1* and *GRIA2* are subunits of the AMPA glutamate receptor, which is responsible for the majority of fast, excitatory, synaptic signaling. *GRIN1* is a subunit of the NMDA receptor, a glutamate receptor implicated in synaptic plasticity, learning, and memory functions. There was a significantly higher expression of all three glutamate receptor subtypes in neurons compared to oligodendrocyte cells (*p* < 0.0001 for all), where no detection was observed for *GRIA2* or *GRIN1* in oligodendrocytes.

#### 3.3.4 Cytotoxicity

**Figure 10:**
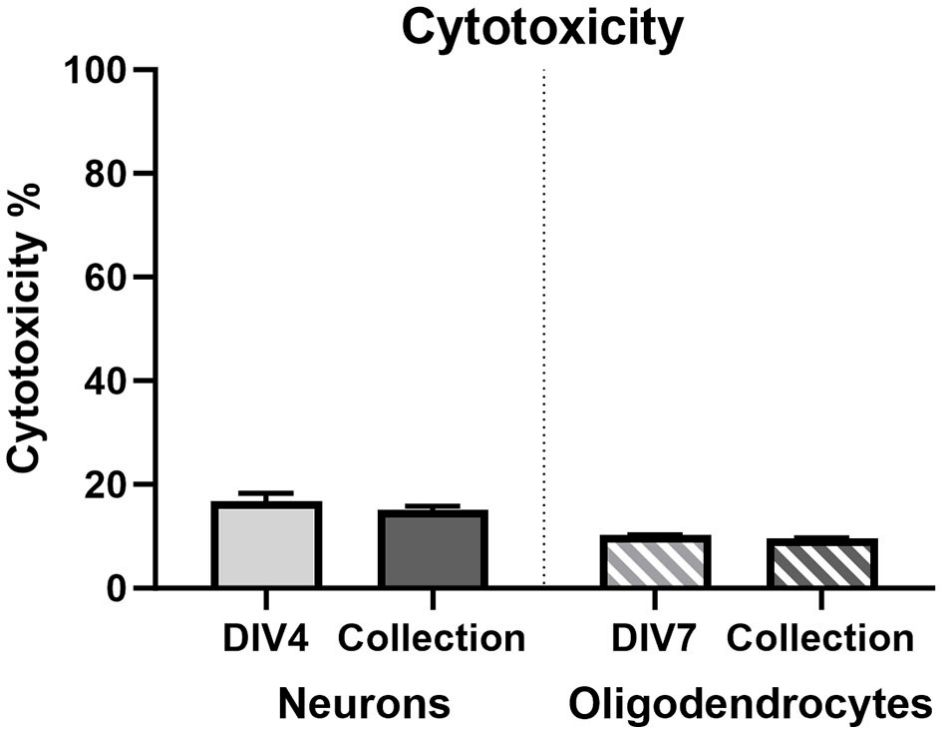
Lactate dehydrogenase cytotoxicity % in primary neurons on DIV3 (light gray bar; *n* = 15) and DIV7 (dark gray bar; *n* = 15) and in primary oligodendrocytes on DIV7 (light gray striped bar; *n* = 15) and DIV13 (dark gray striped bar; *n* = 15).

Lactate dehydrogenase assay (LDH) was used as a measure of cytotoxicity (Figure 10). No statistically significant changes were observed in either cellular population at both time points assessed.

## 4 Discussion

Understanding the mechanisms underlying neurological abnormalities is a critical component of neuroscience research, and the development of clinically relevant and reproducible techniques is crucial for its ongoing advancement. This study presents a novel approach to standard neuron and oligodendrocyte primary cell culture by using the clinically relevant guinea pig as a tissue source. Additionally, this protocol presents the added benefit of the dual isolation of two key cell types from the same tissue sample, allowing for comparison between these major systems. This protocol was developed as an expansion and combination of the classical primary neuronal isolation technique developed in altricial models (Kaplan et al., 1986; Brewer and Cotman, 1989), with the shaking method for isolating oligodendrocytes (McCarthy and de Vellis, 1980). These protocols had previously been refined using rodent models due to the challenges associated with procuring such cells from other species (Chen et al., 2007). To the best of our knowledge, this is the first published method of the isolation of neurons and oligodendrocytes from the guinea pig tissue.

Fetal GA62 guinea pigs were chosen for this protocol due to the trajectory of neurodevelopment occurring at this stage of gestation. In the guinea pig, the period of cortical neurogenesis begins ~20 days after conception, with approximately half of all neuronal maturation and dendritic formation occurring before birth. Additionally, gliogenesis occurs as neurogenesis begins to the end at ~50 days after conception (Kalusa et al., 2021). The guinea pig brain is well developed at birth, with an appropriate degree of cortical folding occurring, and myelination commencing before birth (Hatakeyama et al., 2017). The physiological relevance of this model for the study of perinatal insult is further enhanced by selecting the GA62 timepoint as it coincides with neuronal maturation and the development and expansion of glial cells. This model presents a novel method to study these perinatal processes as altricial species, such as rats and mice, experience many of these developmental processes after birth (Zeiss, 2021). An estimation was made that altricial species between post-natal day 1–10 is equivalent to late gestation brain maturation in humans (Clancy et al., 2007), making the guinea pig an appropriate model for neurodevelopment research. Altricial species present their own benefits, with the gestation of the mouse only 20 days compared to the 70 days of the guinea pig (Murray et al., 2010). This finding makes *in vivo* studies in the guinea pig time-consuming and also makes the studies presented here an attractive alternative. Additionally, altricial species typically have larger litter sizes, making it an accessible tool in neuroscience research. In the case of neurodevelopmental research, however, the physiological relevance of the guinea pig may be more appropriate.

Several adaptions were made to facilitate the growth of cells in this guinea pig model. First, this model was developed to serve as a relatively simple and reproducible technique that utilizes standard laboratory reagents and equipment. Techniques, such as flow cytometry and immunoselection, were avoided in favor of more widely accessible techniques. Neurons were initially grown in a serum-rich medium for 24 h; however, this was removed for the remainder of the experiments due to the promotion of glial cells. The use of cytotoxic, mitotic inhibitors, such as cytosine arabinoside (AraC), was avoided due to interference with potential downstream applications, such as insult treatments that were performed as part of a larger study of perinatal brain injury. Therefore, neuronal cultures had some limited astrocyte growth as evidenced by minor GFAP mRNA expression in these cultures. However, we do not believe that this limited growth affects the quality of the culture as it may be more physiologically relevant when modeling the developing brain.

The development of the oligodendrocyte protocol also posed challenges, as these cells did not respond to protocols that had been developed in other species. The process of developing the oligodendrocyte protocol is possible that, due to the developmental timepoint of these cells, they have an increased susceptibility to damage, including the enzymatic digestion with trypsin, which is typically used in the protocol. Therefore, the use of a more sensitive digestion agent, papain, was chosen, resulting in increased cell survival. A media mix containing a cocktail of specific growth hormones (EGF, FGF, and PDGF) was used to promote OPC expansion in oligodendrocyte cultures. These growth factors synergistically promote the proliferation and survival of OPCs *in vitro* (Yang et al., 2016). Following this expansion, these growth factors were withdrawn from the media as they inhibit differentiation into mature oligodendrocytes. Instead, thyroid hormone (T3) was added to further facilitate this terminal differentiation through the inhibition of cell cycle division (Richardson, 2009). This technique allowed for a reasonably pure population, as shown by the relative absence of GFAP gene expression. It is possible to redirect this oligodendrocyte development to astrocyte development through the inclusion of serum and withdrawal of all growth factors, a technique that may be of interest for some investigations.

This model was validated through the combination of stage-specific mRNA markers and immunocytochemistry staining. Both neuronal populations displayed a clear expression of key neuronal markers, including those specific to GABAergic neurons (*SST, CALB, PVALB*). Glutamatergic activity also appears to be present, as neuronal cultures exhibit the expression of vesicular glutamatergic transporters VGLUT2 and VGLUT3, along with the glutamatergic synapse marker DLG4. Additionally, neurons displayed MAP2 and NeuN staining, a marker of neuronal dendrites. The purity of the neuronal culture appears to be primarily neuronal, with very few cells not displaying positive NeuN staining. Oligodendrocyte cell populations showed minimal neuronal gene expression and, instead, had strong expression for all key markers of the oligodendrocyte lineage pathway (*OLIG2, NCAM1, CSG4, GalC, MBP*). Oligodendrocyte cultures also showed primarily oligodendrocyte, with very few cells not displaying positive OLIG2 staining, and minimal gene expression for other population markers. The timepoint that oligodendrocyte cultures were collected was chosen as earlier timepoints exhibited a far higher expression of early OPC markers, such as *NCAM1* and *CSPG4*. Based on morphology and relative gene expression, these cells appear to be primarily at the immature oligodendrocyte stage, making them an excellent model to investigate maturation-related developmental changes.

The receptor expression found on these cells further highlights the potential uses of this cell culture model. Oligodendrocyte cells displayed D2 receptor expression and an absence of D1, which validates what has previously been found *in vitro* and *in vivo* as D2 receptors have been suggested to influence oligodendrocyte maturation (Bongarzone et al., 1998). In terms of GABAA receptor subunit expression, neuronal cells displayed the expression of the four subunits examined. Oligodendrocytes exhibited the expression of GABRA3 and minimal GABRA2, which again is a representative of what has been previously found in a neonatal rat model (Ordaz et al., 2021).

The key glutamatergic receptor subunits GRIA1 and GRIA2, which feature in AMPA receptors, and the NMDA receptor GRIN1 subunit were all found to be expressed in the neuronal culture population. The oligodendrocyte culture population in this study displayed the expression of the calcium permeable GRIA1 subunit but not the calcium impermeable GRIA2 subunit. Additionally, GRIN1 expression was also absent. AMPA-mediated calcium signaling has been found to be integral in the proliferation of oligodendrocytes while NMDA receptor-mediated calcium signaling is typically associated with the differentiation of maturing oligodendrocytes (Paez and Lyons, 2020). The differential expression of calcium permeable AMPA receptors and negligible expression of NMDA receptor subunits in this oligodendrocyte population further implicate this population as immature, proliferating oligodendrocytes.

This protocol only describes the culture of neurons from the frontal cortex of GA62 guinea pigs. The frontal cortex region was chosen as it is a key area of the brain often implicated in neurodevelopmental disorders, such as attention deficit hyperactive disorder (ADHD) (Arnsten, 2009). The study of perinatal insults would further benefit from the ability to investigate multiple brain regions—an idea Cohen et al. (2000) also have employed. Cohen et al. (2000) derived neuronal cultures from the frontal cortex, hippocampus, and cerebellum from the same brain of fetal guinea pigs. Our work with this protocol will continue to examine derivation of neurons and oligodendrocytes from multiple regions within the same brain. Progress has been made in sustaining cerebellar cultures from GA62 fetal guinea pigs using a slightly modified protocol. This brain region is chosen for its high affinity for damage following perinatal insult (Kwan et al., 2015; Benterud et al., 2018). We further suggest that this protocol could further be adapted to establish cultures from other brain regions such as the hippocampus, further decreasing tissue wastage and improving the relevance of this protocol for investigating perinatal insults that often affect these areas.

As this is the first-time primary neurons and oligodendrocytes have been isolated from guinea pig brain tissue, there are limitations in the study that need to be addressed. The guinea pig has a longer gestation than other rodent species (70 days) and required time-mating to be performed to collect cells from fetuses of the same gestational age (Hirst et al., 2018). Therefore, this finding limits the ease at obtaining samples and puts limits on the number of experiments that can be performed. However, as the cells come from tissue that has a much more similar developmental trajectory to humans, we believe that this warrants the reduced number of experiments that can be performed while still allowing for a sufficient number of cells to conduct necessary downstream experiments. Additionally, we included *n* = 15 for each cell type, ensuring that the protocol was consistent and reproducible. Future research would benefit from further immunocytochemistry characterization of specific markers that are exhibited by these cellular populations. For example, the characterization of neuronal circuitry and calcium activity in these cellular populations would be a useful addition.

In conclusion, this study has described a novel protocol for the isolation of key cells from guinea pig brain tissue. The establishment and validation of primary neuronal and oligodendrocyte cultures from the same tissue source provide a unique opportunity to investigate neurodevelopment and associated pathologies in a model more clinically relevant to human perinatal neurodevelopment.

## Data availability statement

The raw data supporting the conclusions of this article will be made available by the authors, without undue reservation.

## Ethics statement

The animal study was approved by University of Newcastle Ethics Committee. The study was conducted in accordance with the local legislation and institutional requirements.

## Author contributions

RM: Conceptualization, Data curation, Formal analysis, Investigation, Methodology, Validation, Writing – original draft, Writing – review & editing. CP: Conceptualization, Methodology, Writing – review & editing. RK: Conceptualization, Methodology, Writing – review & editing. HP: Conceptualization, Funding acquisition, Supervision, Validation, Writing – review & editing. JH: Funding acquisition, Supervision, Validation, Writing – review & editing. JS: Conceptualization, Funding acquisition, Methodology, Project administration, Resources, Supervision, Validation, Visualization, Writing – review & editing.
